# Relation of the *pdxB-usg*-*truA*-*dedA* Operon and the *truA* Gene to the Intracellular Survival of *Salmonella enterica* Serovar Typhimurium

**DOI:** 10.3390/ijms20020380

**Published:** 2019-01-17

**Authors:** Xiaowen Yang, Jiawei Wang, Ziyan Feng, Xiangjian Zhang, Xiangguo Wang, Qingmin Wu

**Affiliations:** 1 Animal Science and Technology College, Beijing University of Agriculture, Beijing 102206, China; yangxiaowen@icdc.cn (X.Y.); wangjiawei20190110@gmail.com (J.W.); zy.feng0215@gmail.com (Z.F.); 2 Key Laboratory of Animal Epidemiology and Zoonosis of the Ministry of Agriculture, College of Veterinary Medicine, China Agricultural University, Beijing 100193, China; 3 State Key Laboratory for Infectious Disease Prevention and Control, Collaborative Innovation Center for Diagnosis and Treatment of Infectious Diseases, National Institute for Communicable Disease Control and Prevention, Chinese Center for Disease Control and Prevention, Beijing 102206, China; 4 Tianjin Xiqing District Animal Husbandry and Aquatic Industry Development Service Center, Tianjin 300380, China; zhangxiangjian2019@gmail.com

**Keywords:** *usg*, *truA*, *Salmonella enterica* serovar Typhimurium, oxidative stress, intracellular survival

## Abstract

*Salmonella* is the genus of Gram-negative, facultative intracellular pathogens that have the ability to infect large numbers of animal or human hosts. The *S. enterica usg* gene is associated with intracellular survival based on ortholog screening and identification. In this study, the λ-Red recombination system was used to construct gene deletion strains and to investigate whether the identified operon was related to intracellular survival. The *pdxB*-*usg*-*truA*-*dedA* operon enhanced the intracellular survival of *S. enterica* by resisting the oxidative environment and the *usg* and *truA* gene expression was induced by H_2_O_2_. Moreover, the genes in this operon (except for *dedA*) contributed to virulence in mice. These findings indicate that the *pdxB*-*usg*-*truA*-*dedA* operon functions in resistance to oxidative environments during intracellular survival and is required for in vivo *S. enterica* virulence. This study provides insight toward a better understand of the characteristics of intracellular pathogens and explores the gene modules involved in their intracellular survival.

## 1. Introduction

*Salmonella* is a genus of Gram-negative and facultative intracellular pathogens that consists of a large group of genetically similar organisms with the ability to infect many animal and human hosts [1]. In 2005, the *Salmonella* genus was divided into *S. bongori* and *S. enterica* species [2]. *Salmonella* spp. are the second most common causative-agents of gastrointestinal infections in humans, after *Campylobacter* spp. [3]. Some *Salmonella* serovars cause large outbreaks of gastroenteritis associated with contaminated meat and produced or processed food [4].

A previous study found that the *S. enterica usg* gene was associated with intracellular survival based on ortholog screening and identification [5]. Because the complemented STΔusg/p-usg and wild type strains showed significant variation in the infection assay, our lab suggested that the *usg* gene was located in an operon with other genes. Operon prediction for the *S. enterica* strain LT2 whole genome sequence [6] found that the *pdxB*, *usg*, *truA* and *dedA* genes were located in the same operon. The promoter of this operon was located upstream of the *pdxB* gene (http://www.softberry.com/berry.phtml?topic=bprom&group=programs&subgroup=gfindb; accessed on 2 May 2018). Based on the genome annotation, the *pdxB* gene encodes 4-phosphoerythronate dehydrogenase, which is associated with de novo vitamin B6 biosynthesis [7]; the *usg* gene encodes putative aspartate-semialdehyde dehydrogenase, *truA* encodes RNA pseudouridine (38–40) synthase, and the *dedA* gene encodes a hypothetical protein.

In this study, the λ-Red recombination system was used to construct gene deletion strains and investigate whether the identified operon was related to intracellular survival. Furthermore, this study investigated the possible functions of the genes in the operon and assessed their roles in the virulence of *S. enterica*.

## 2. Results

### 2.1. The pdxB-usg-truA-dedA Operon Is Required for Intracellular Survival of S. enterica

**Figure 1:**
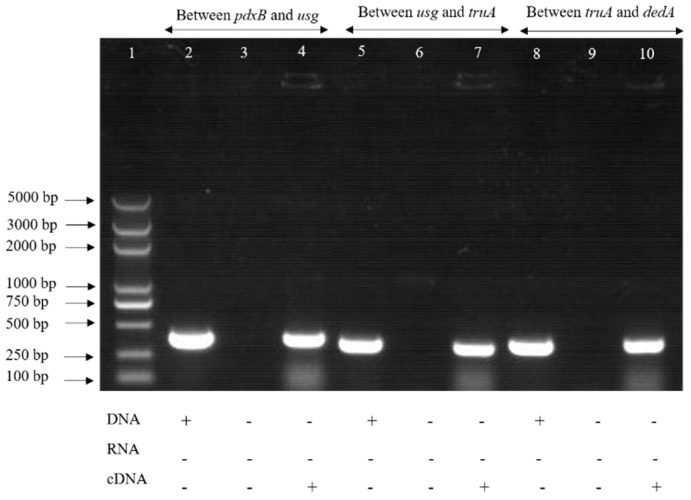
Analysis of co-transcription detection in the operon. Note: “+” means there was a fragment, “−” means there was no fragment. Lane 1 was a DNA marker, lanes 2, 5 and 8 were amplified using genome DNA; lanes 3, 6 and 9 were amplified using total RNA; lanes 4, 7 and 10 were amplified using cDNA.

The predicted operon showed that the *pdxB*, *usg*, *truA* and *dedA* genes were transcribed in the same direction, indicating that these genes may have co-transcription. The primers were used to amplify the cDNA of the upstream and downstream genes and the intergenic regions, and the DNA and RNA were used as controls. The results revealed that the genes in the operon were co-transcribed. The above results indicate that the operon contains four genes: *pdxB*, *usg*, *truA* and *dedA* (Figure 1). The expression of each gene, using RT-PCR, showed that the genes in the operon had no effect on other genes in the lysogeny broth (LB) medium.

**Figure 2:**
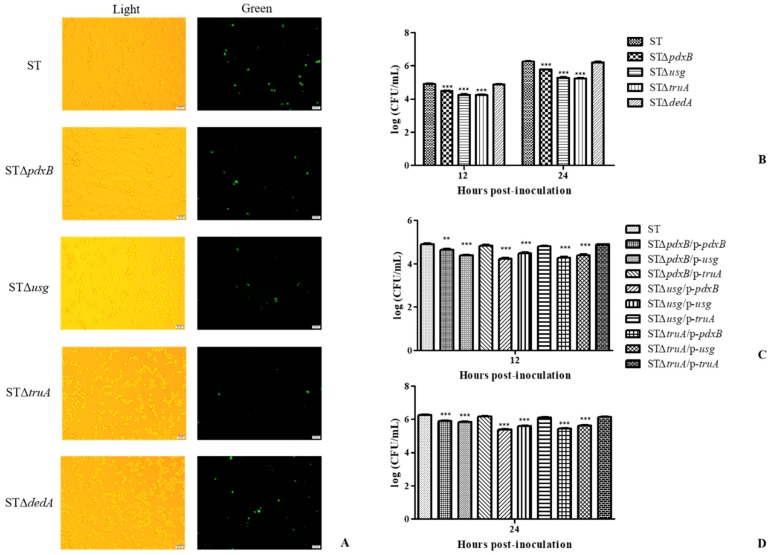
Intracellular survival of the operon gene deletion mutants and complemented strains in macrophages. (**A**) The infection process in J774A.1 macrophages by the strains was observed under a microscope at 12 h post-infection (40×). “Light” indicates that the cells were observed under natural light, and “Green” indicates that the cells were under a fluorescence microscope. (**B**) Bacterial loads of the gene deletion strains at 12 and 24 h post inoculation. (**C**,**D**) Bacterial loads of the complemented strains with different plasmids at 12 and 24 h post-inoculation. ** *p* < 0.01, *** *p* < 0.001.

The *pdxB*, *usg*, *truA* and *dedA* genes were located in the same operon and deletion strains for these genes were constructed using the λ-Red recombination system. The intracellular survival of the gene deletion strains was assessed using J774A.1 macrophage cells (Figure 2). At 12 and 24 h post-infection, the cells infected with the STΔ*pdxB*, STΔ*usg* and STΔ*truA* strains showed lower bacterial loads than the cells infected with the ST and STΔ*dedA* strains (*p* < 0.01) (Figure 2A,B). The STΔ*pdxB*, STΔ*usg* and STΔ*truA* strains had reduced replication abilities inside the J774A.1 macrophages and, therefore, exhibited reduced virulence in vitro. However, the STΔ*dedA* strain did not show reduced replication.

Subsequently, the complemented gene plasmids were electroporated into all of the gene deletion mutants. As shown in Figure 2C,D, the STΔ*pdxB* strains electroporated with the recombinant plasmids p-*pdxB* and p-*usg* showed significant differences in bacterial loads (*p* < 0.001), whereas the STΔ*pdxB* strains electroporated with the recombinant plasmid p-*truA* had almost the same bacterial loads in the macrophages. Similar results were found for the other complemented strains. These results indicated that the *truA* gene played a key role related to intracellular survival in this operon.

In summary, these results showed that all genes in this operon, except for the *dedA* gene, confirmed that *S. enterica* has the ability to survive in macrophages and that the *truA* gene played a key role in this function.

### 2.2. The pdxB-usg-truA-dedA Operon Contributes to Virulence in Mice

**Figure 3:**
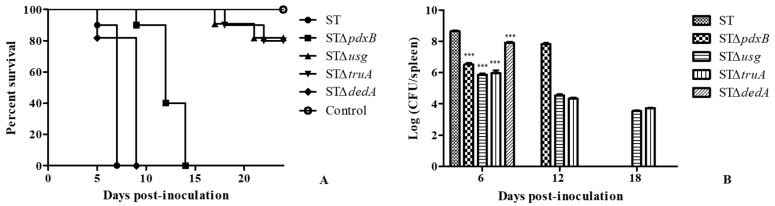
Survival of the gene deletion mutant and wild type strains in mice. (**A**) Survival curves of the gene deletion and wild type strains. (**B**) Bacterial loads of the spleens at six, 12, and 18 days post-inoculation with the gene deletion and wild type strains. *** *p* < 0.001.

Ten mice were intraperitoneally inoculated with a dose of 10^5^ Colony-Forming Units (CFU) of the STΔ*pdxB*, STΔ*usg*, STΔ*truA*, STΔ*dedA* and wild type strains. The animals did not survive more than seven days after intraperitoneal inoculation with the wild type strain and not more than nine days post-inoculation with STΔ*dedA*. The mice in the group inoculated with STΔ*pdxB* did not survive more than 15 days. Conversely, the survival rates of the groups inoculated with STΔ*usg* and STΔ*truA* were both 80% at 24 days post-inoculation (Figure 3A).

Five infected mice from each group were randomly selected at 6, 12, and 18 days post-inoculation. Their spleens were removed to assess the bacterial loads (Figure 3B). At six days post-inoculation, the bacterial loads were significantly lower for the gene deletion strains than for the wild type strain and a large reduction (above 2-log) in the spleen bacterial load was observed in the mice inoculated with STΔ*usg* and STΔ*truA* compared to the mice infected with the wild type strains. At 12 days post-inoculation, the spleen bacterial load increased in the mice inoculated with STΔ*pdxB*, which was similar to the load measured in the mice inoculated with the wild type strain at six days. The bacterial loads of the mice inoculated with STΔ*usg* and STΔ*truA* reduced slowly (Figure 3B). The results showed that *usg* and *truA* contributed to virulence in mice, which was consistent with the cell infection assay results.

### 2.3. The usg and truA Expression Levels Were Higher in the Oxidative Environment

**Figure 4:**
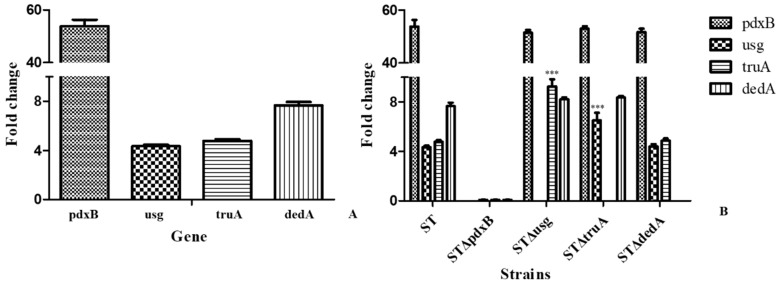
Gene expression levels in the bacterial strains. (**A**) Gene expression levels in the wild type strain under oxidative conditions. (**B**) Gene expression levels in wild type and gene deletion strains under oxidative conditions. *** *p* < 0.001.

The expression levels of genes in the operon were evaluated in the gene deletion and wild type strains in the routine growth medium and under oxidative conditions (Figure 4). As shown in Figure 3A, *pdxB*, *usg*, *truA* and *dedA* expression was induced in the wild type strain by H_2_O_2_ and was significantly higher in the samples treated with H_2_O_2_ than in the untreated controls (Figure 4A).

Under oxidative conditions, the *usg*, *truA* and *dedA* genes were barely expressed when *pdxB* was deleted compared with the expression levels in the wild type strain. The *pdxB* gene expression levels were similar in the *usg*, *truA* and *dedA* gene deletion strains. When *dedA* was deleted, the expression levels of the other genes were not significantly different from the expression levels in the wild type strain. When *usg* was deleted, *truA* expression was induced by oxidative conditions and *truA* expression was significantly higher in these strains compared with the wild type strain. *Usg* expression also significantly increased when *truA* was deleted (Figure 4B).

### 2.4. The pdxB-usg-truA-dedA Operon Contributed to Resistance to Oxidative Conditions

**Figure 5:**
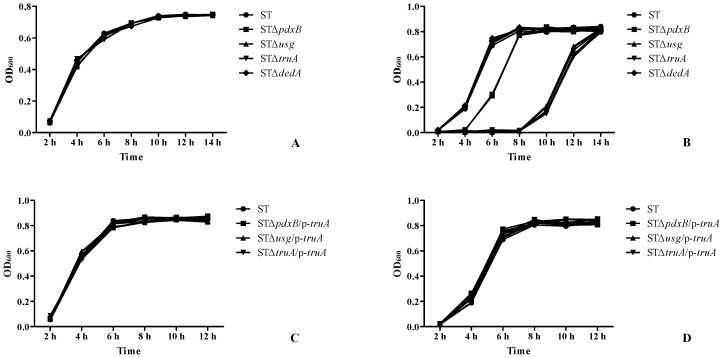
Growth characteristics of the gene deletion and complemented strains in the lysogeny broth (LB) medium and under oxidative conditions. (**A**) Growth characteristics of the gene deletion strains in the LB medium. (**B**) Growth characteristics of the gene deletion strains under oxidative conditions. (**C**) Growth characteristics of the strains complemented with the p-*truA* plasmid in the LB medium. (**D**) Growth characteristics of the strains complemented with p-*truA* plasmid under oxidative conditions.

The growth characteristics of the gene deletion and parent strains were determined in an LB medium. No significant variations were observed between the gene deletion and wild type strains (Figure 5A). These results suggested that the genes in the operon did not affect the in vitro growth of *Salmonella* spp. at normal temperatures. Under oxidative conditions, all of the strains grew slowly for the first 2 h. After 6 h, the growth of the wild type and STΔ*dedA* strains reached the plateau phase (Figure 3B). At this time, the STΔ*pdxB* strain was in the logarithmic phase, and the STΔ*usg* and STΔ*truA* strains had barely replicated. At 10 h post-infection, the STΔ*usg* and STΔ*truA* strains began to replicate, whereas the other strains were in the plateau phase (Figure 5B).

The growth characteristics and oxidative resistance were also assessed for the STΔ*pdxB*/p-*truA*, STΔ*usg*/p-*truA* and STΔ*truA*/p-*truA* strains, which were complemented strains with the same recombinant p-*truA* plasmid (Figure 3C,D). These strains had characteristics similar to the wild type, even under oxidative conditions. All of the results suggested that the operon (except for the *dedA* gene) contributed to a resistance to oxidative conditions promoted the survival of *S. enterica* in macrophages.

## 3. Discussion

*S. enterica* is a common facultative intracellular pathogen. The main pathway used by macrophages to eliminate invading pathogens are endocytosis and digestion. *S. enterica* can exploit multiple aspects of host defenses to promote its replication in the host after adaptation to a variety of harsh environments, such as oxidative conditions [8]. A previous study found that the *usg* gene was related to intracellular survival and that the *pdxB*, *usg*, *truA* and *dedA* genes were located in the same operon. Another study found that *pdxB* was related to intracellular survival in *S. enterica*. This study found that a *pdxB* mutant strain was sensitive to oxidative conditions and reduced the bacterial load in macrophages. Another study found that *pdxB* in *E. coli* and *Pseudomonas aeruginosa* contained tightly bound NAD^+^ and/or NADH [7,9,10] and that the nucleotide-binding domains of *pdxB* were homologous to the corresponding domains of d-3-phosphoglycerate dehydrogenase (PGDHs) from *E. coli* and *Mycobacterium tuberculosis* [7]. Because orthologs usually have conserved biological structures and functions [11,12], the results of this study suggest that *pdxB* in *S. enterica* has the same function. The *usg* and *truA* genes had similar results in the cell assay, similar expression levels under oxidative conditions, and inhibited TNF-α and IL-1β expression in macrophages. One study used random insertions of TnphoA-132 and found that *truA* was one target of glyoxal [13]. Another study showed that *truA* was associated with resistance to quinoxaline 1, 4-dioxides (QdNOs) in *E. coli*, which have been used in animals as antimicrobial agents and growth promoters for decades [14]. The results showed that *usg* and *truA* were related to intracellular survival. Although the *dedA* gene was in the same operon as the other genes, no effect on intracellular survival was observed for this gene.

There were many genes related to intracellular survival via oxidative resistance. The *sodA* gene deletion strain resulted in a slightly reduced growth rate, low SOD activity, increased susceptibility to reactive oxygen species and chicken serum, and no effect on the motility of the wild type strain [15]. One study found that three catalases (*KatE*, *KatG*, and *KatN*) and two alkyl hydroperoxide reductases (*AhpC* and *TsaA*) were related to oxidative resistance using silico genome analysis and gene deletion methods [16]. Large-scale profiling of *Salmonella* protein expression was performed under H_2_O_2_ treatment. The results showed that the abundance of 116 proteins were altered significantly among 1600 quantified proteins and that iron acquisition systems were induced to promote bacterial survival under oxidative stress [17]. Macrophages play important roles in the phagocytosis of pathogens and antigen presentation. Macrophages are immediately activated after phagocytosing pathogens, resulting in a variety of bactericidal mechanisms. These mechanisms include both oxidative and non-oxidative bactericidal mechanisms [8,18]. These poor survival environments lead *Salmonella* spp. to secrete effectors and generate a replicative compartment known as the Salmonella-containing vacuole (SCV). This study showed that genes in this operon (except for the *dedA* gene) conferred *S. enterica* with the ability to survive in macrophages and that the *truA* gene played a key role. The *usg* and *truA* expression levels were increased under oxidative treatment. All of these results suggested that this operon enhanced the intracellular survival of *S. enterica* by increasing resistance to oxidative environments and that *truA* played a key role in this function.

Operons are polycistronic clusters of genes transcribed from a promoter at the 5′ end of the cluster [19]. Several operons reportedly related to virulence have been identified in *S. enterica* [20,21,22,23,24]. Typically, genes in the same operon have similar functions and interact with each other. The amino acid sequences *pdxB*, *usg*, *truA* and *dedA* were uploaded to the STRING database [25] to predict protein–protein interactions. *Usg* and *truA* had the highest combined association score (0.935). Co-expression of *usg* and *truA* orthologs was also found in *Acinetobacter* sp. ADP1 and *Pseudomonas aeruginosa*. The combined association score of *dedA* was lower than the association scores for the other genes and no co-expression of *dedA* orthologs had been found to date. The results of our study are similar to our predictions.

## 4. Materials and Methods

### 4.1. Ethics Statement

All animal research was approved by the Beijing Association for Science and Technology. The approval ID is SYXK (Beijing) 2015–0028 (Validity period: 22 September 2015 to 22 September 2020), and the animal research complied with the Beijing Laboratory Animal Welfare and Ethics guidelines of the Beijing Administration Committee of Laboratory Animals.

### 4.2. Bacterial Strains and Media

All of the bacterial strains and plasmids used in this study are listed in Table 1. The *S. typhimurium* and *E. coli* strains, including the parental strain and the derived mutants, were routinely grown or incubated in an LB medium. Antibiotics were added at the following concentrations when required: ampicillin, 100 mg/L and chloramphenicol, 34 mg/L. All bacterial strains were frozen at −80 °C with 15–20% (*v*/*v*) glycerol.

### 4.3. Mice

BALB/c mice (aged 4 to 6 weeks) were purchased from the Weitong Lihua Laboratory Animal Services Center (Beijing, China), and bred in individually ventilated cage rack systems. All experiments involving animals followed the regulations of the Beijing Administration Office for Laboratory Animals.

### 4.4. Construction of Gene Deletion and Complemented Gene Deletion Mutant Strains

Deletion mutants and their complemented mutants were constructed for all genes in the operon. Gene deletion mutants were constructed using the λ-Red recombination system. After sequencing confirmation, the recombinant plasmids with the coding regions and their promoters were subsequently electroporated into every gene deletion mutant to complement the gene function. The complemented strains were selected from an LB medium containing ampicillin. The primers are shown in Table S1. The gene deletion and complemented gene deletion mutants were confirmed by PCR amplification and sequencing.

Total RNA was extracted from all strains using TRIzol (Invitrogen, Inc., Carlsbad, CA, USA) according to the manufacturer’s instructions and treated with DNase (TaKaRa Bio, Inc., Dalian, China) before reverse transcription to remove DNA contamination. Total RNA was dissolved in diethypyrocarbonate (DEPC)-treated water, and the concentration and purity of the total RNA were estimated by reading the absorbance at 260 and 280 nm, respectively. cDNA was synthesized using the Prime Script^TM^ RT Reagent Kit (TaKaRa Bio, Inc., Dalian, China), according to the manufacturer’s instructions. The reverse transcription product was stored at −20 °C. PCR was performed with the primers shown in Table S1 to evaluate gene expression.

To determine if the genes in the operon were co-transcribed, the intergenic regions of genes were amplified by RT-PCR [27]. The RNA of the parental strain was extracted and cDNA was synthesized by reverse transcription using the method descibed before. The gene spacers were amplified using primers (Table S1) while the wild strain genomic DNA and RNA were used as controls. The amplification system and conditions were the same as before.

### 4.5. Cell Infection Assay

To investigate the intracellular survival of the strains, infection assays were performed using J774A.1 murine macrophages (Key Laboratory of Animal Epidemiology and Zoonosis of the Ministry of Agriculture, Beijing, China). The cells were cultured in 24-well plates and infected with each strain at a multiplicity of infection (MOI) of 10 CFU. Then, the infected plates were centrifuged at 1000 rpm for 5 min at room temperature and incubated at 37 °C in an atmosphere containing 5% (*v*/*v*) CO_2_. After 20 min, the cells were washed three times with phosphate buffered solution (PBS) and incubated in a medium containing gentamycin (50 μg/mL) at 37 °C under 5% CO_2_ until the end of the infection period. At 12 and 24 h post infection (p.i.), the cells were washed and lysed, and the numbers of bacteria exhibiting intracellular survival were determined through serial dilution and plating on an LB medium.

The pBR322 plasmid encoding the green fluorescent protein (GFP) was transferred into the parent and gene deletion strains using the electroporation method. Recombinant clones were selected from the LB medium containing ampicillin. Then, infection assays were performed as described above. After 12 h of incubation, the macrophages were washed three times with PBS. Infection of the J774A.1 macrophages by the strains was observed under a fluorescence microscope (Olympus, Tokyo, Japan).

### 4.6. Growth Characteristics and Oxidative Resistance Assay

The in vitro growth analysis of the deletion mutants and complemented strains was described previously. An oxidative resistance assay was performed as follows. One colony of each strain was inoculated into 3 mL of LB or LB with ampicillin medium and cultured overnight at 37 °C with shaking at 200 rpm. Subsequently the cultures were adjusted to the same concentration (OD_600_ ≈ 1.0) and a 50 μL sample of each strain was inoculated into 5 mL of an LB or LB with ampicillin medium. Then, 30% H_2_O_2_ was add to the liquid medium (final concentration 4.4 mM) [28] to provide an oxidative environment. The cultures were incubated at 37 °C with shaking at 200 rpm, and the OD_600_ value was determined every 2 h using a BioTek microplate reader (Gene Company Limited, Hong Kong, China).

### 4.7. Gene Expression Levels in an Oxidative Environment

The gene expression levels under oxidative treatment were assessed by real-time PCR (RT-PCR). One colony of the gene deletion and parent strain was inoculated into 3 mL of LB medium and cultured overnight at 37 °C with shaking. The cultures were adjusted to the same concentration (OD_600_ ≈ 1.0). A 50 μL sample of each strain was inoculated into 5 mL of LB medium and LB medium with H_2_O_2_. Total RNA was extracted from all strains, and cDNAs were obtained as described above. The cDNA samples were subjected to quantitative RT-PCR using the SYBR^®^ Premix Ex Taq^TM^ II Kit (TaKaRa Bio, Inc., Dalian, China). Each PCR reaction consisted of 2 μL of cDNA, 0.8 μL of each primer (10 μM), 10 μL of SYBR^®^Premix Ex Taq^TM^ II, and 20 μL RNase-free water. The cycling conditions were a denaturation step, at 95 °C for 10 min, followed by 40 cycles of 95 °C for 10 s and 60 °C for 20 s. The specificity of the RT-PCR products was confirmed using a melting curve analysis. These reactions were repeated in triplicate for every sample as technical replicates. Gene mRNA quantification was performed using the 2^−^^ΔΔ*C*t^ method to analyze the expression levels. The 16S rRNA expression level in *S. enterica* was used as a reference to normalize all values. The results presented in this study represent the averages from at least three separate experiments.

### 4.8. Virulence in BALB/c Mice

There were two experiments. The first experiment concerned the survival of the mice. Ten mice were intraperitoneally inoculated with a dose of 10^5^ CFU of the gene deletion and wild type strains in 100 μL of phosphate-buffered saline (PBS) [29]; the control group included five mice intraperitoneally inoculated with 100 μL of PBS. The survival of the mice was observed over the next 24 days. The second experiment was the virulence of the gene deletion strains. Based on survival time, five mice were intraperitoneally inoculated with the same dose of the gene deletion and wild type strains. Five infected mice from each group were randomly selected at 6, 12, and 18 days post-inoculation. At each time point, the spleens were removed and homogenized individually in an aseptic manner in 1 mL of PBS and then serially diluted to isolate the bacteria. The results are presented as the mean number of CFU per spleen± the standard deviation (SD) in each group.

### 4.9. Statistical Analysis

The statistical analyses of the data, including the data from the growth curve analysis, cell infection study, oxidative resistance assay and virulence experiments, were performed using IBM SPSS Statistics version 23 (IBM, Armonk, New York, NY, USA, https://www.ibm.com/analytics/data-science/predictive-analytics/spss-statistical-software). A *p* value < 0.05 obtained through one-way analysis of variance (ANOVA) was considered significant. All graphics were drawn with GraphPad Prism 5 (GraphPad Software, La jolla, CA, USA, https://www.graphpad.com/).

## 5. Conclusions

In conclusion, this study used the λ-Red recombination system to construct gene deletion strains and determine whether the identified operon was related to intracellular survival. Except for the *dedA* gene, all of the genes in this operon confirmed the ability of *S. enterica* to survive in macrophages, and the *truA* gene played a key role in resistance to oxidative conditions. Moreover, the genes in this operon (except for *dedA*) contributed to virulence in mice. These findings indicate that the *pdxB*-*usg*-*truA*-*dedA* operon functions in resistance to oxidative environments and contributes to intracellular survival; moreover, the operon is required for the virulence of *S. enterica* in vivo. In this study, clues were examined to gain a better understanding of the characteristics of intracellular pathogens and to explore the gene modules involved in the intracellular survival of intracellular pathogens.

## Figures and Tables

**Table 1:** Strains and plasmids used in this study.

Strains or Plasmids	Description/Purpose	Source or Reference
Strains		
*S. typhimurium* ATCC14028 (ST)	Wild type (WT)	Guangdong Culture Collection Center
STΔ*pdxB*	Δ*pdxB* mutant of ST by the λ-Red recombination system	This study
STΔ*usg*	Δ*usg* mutant of ST by the λ-Red recombination system	Our lab
STΔ*truA*	Δ*truA* mutant of ST by the λ-Red recombination system	This study
STΔ*dedA*	Δ*dedA* mutant of ST by the λ-Red recombination system	This study
STΔ*pdxB*/p-*pdxB*	STΔ*pdxB* harboring the pBR322-*pdxB* plasmid, complement strains	This study
STΔ*pdxB*/p-*usg*	STΔ*pdxB* harboring the pBR322-*usg* plasmid, complement strains	This study
STΔ*pdxB*/p-*truA*	STΔ*pdxB* harboring the pBR322-*truA* plasmid, complement strains	This study
STΔ*usg*/p-*pdxB*	STΔ*usg* harboring the pBR322-*pdxB* plasmid, complement strains	This study
STΔ*usg*/p-*usg*	STΔ*usg* harboring the pBR322-*usg* plasmid, complement strains	Our lab
STΔ*usg*/p-*truA*	STΔ*usg* harboring the pBR322-*truA* plasmid, complement strains	This study
STΔ*truA*/p-*pdxB*	STΔ*truA* harboring the pBR322-*pdxB* plasmid, complement strains	This study
STΔ*truA*/p-*usg*	STΔ*truA* harboring the pBR322-*usg* plasmid, complement strains	This study
STΔ*truA*/p-*truA*	STΔ*truA* harboring the pBR322-*truA* plasmid, complement strains	This study
DH5α	For cloning	Takara
Plasmids		
pKD3, pKD46 and pCP20	λ-Red recombination system	Datsenko and Wanner, 2000 [26]
pBR322	For constructed complement strains	Virulent laboratory
p-*pdxB*	*pdxB* of ST product cloned into pBR322 for complementation assay	This study
p-*usg*	*usg* of ST product cloned into pBR322 for complementation assay	Our lab
p-*truA*	*truA* of ST product cloned into pBR322 for complementation assay	This study

## Supplementary Materials

Supplementary materials can be found at http://www.mdpi.com/1422-0067/20/2/380/s1.
