# Supplementary material for: Relation of the pdxB-usg-truA-dedA Operon and the truA Gene to the Intracellular Survival of Salmonella enterica Serovar Typhimurium
Source: Int J Mol Sci. 2019 Jan 17;20(2):380. doi: 10.3390/ijms20020380 (PMC6358828; doi:10.3390/ijms20020380)
Supplement: Supplementary file 1 [file ijms-20-00380-s001.pdf]

Table S1. Primers used in this study.

Name	Sequence (Restriction Enzyme Used) (5′-3′)	Locus (Gene)
	**Primers used for construction of the gene mutants**	
wu3574	TTAACGGCTGACATGGGAATTAGC	cat^r
wu3575	AGCGATTGTGTAGGCTGGAG	
wu4015	GCGAACAGACCGCTGGAA	*pdxB* upstream
wu4016	CACAATCGCTGTCTTCTTAATGCTCCCTGT	
wu4017	CAGCCGTTAACGACTGTCGTACCTGTTTGA	*pdxB* downstream
wu4018	GCGTGATTTTGCTGCGCT	
wu4019	TCCAGATAACTACGGCGGA	*truA* upstream
wu4020	CACAATCGCTATCCGAATCGCACACCCG	
wu4021	CAGCCGTTAAGGACATCAGTACAGATACTC	*truA* downstream
wu4022	GCGCCAGGAACGTCGTAT	
wu4023	TTGGCTCCAGCTCGCTAC	*dedA* upstream
wu4024	CACAATCGCTAACTCAGCGGTTCGACCA	
wu4025	CAGCCGTTAATGTCTTTGCCTTTCTACCCG	*dedA* downstream
wu4026	TTACCTCGTTTCGCGCAGT	
	**Primers used for construction of the complement strains**	
wu3957	CTTCAGACATGACTGTCGTACCTGTTTG	promoter
wu3967	CGAAGCTTTCGCAGCGGAAAGAGGA	
wu3961	CGGGATCCGACCAGGCCGGTTTCACA	*truA*
wu3964	ATGTCCGGACAGCAATCATCGGC	
wu4027	CGGGATCCTGGCGTTCAGCCAGTGTT	*pdxB*
wu4028	CGAAGCTTATTACGCCCTTCGCCTGG	
	**Primers used for detect the expression of genes by RT-PCR**	
wu4029	GCAACGCGAAGAACCTTACC	16S rRNA
wu4030	TTCACAACACGAGCTGACGA	
wu4031	CATTCACGGCGCTCAAGATA	*pdxB*
wu4032	ACTGGCGCATTTGGTGTATG	
wu4033	CCGCAATCACGTTACGGTTA	*usg*
wu4034	TGCAGGTTGCCTGGTGATAG	
wu4035	CGGCGCAGAACACGTTAATA	*truA*
wu4036	TGAGTACGACGGCAGCAAGT	
wu4037	TGCCGGACAATCTCAATGAC	*dedA*
wu4038	CGCCGGTTATTTCTTCGGTA	
	**Primers used for co-transcription detection in the operon**	
wu4107	GACCGACTTGCCACCAAAAC	Between pdxB and usg
wu4108	ACTGCGTAAAGTCGCTGGAA	
wu4109	GTGAACGCCGGCATCGGTA	Between usg and truA
wu4110	CCGGCAATCCACAGCTCT	
wu4111	ACAAACAGCAGCGAGTCG	Between truA and dedA
wu4112	AGTAGGCGCTCACAACCA	
